# (*R*)-2-Methyl­piperazine-1,4-diium tetra­chloridoanti­monate(III) chloride

**DOI:** 10.1107/S1600536810047689

**Published:** 2010-11-24

**Authors:** Lu Li, Guo-Xi Wang

**Affiliations:** a Department of Chemical and Environmental Engineering, Anyang Institute of Technology, Anyang 455000, People’s Republic of China

## Abstract

In the complex anion of the title compound, (C_5_H_14_N_2_)[SbCl_4_]Cl, the Sb atom is tetra­coordinate within a saw-horse configuration. The cation adopts a chair conformation. The crystal structure is stabilized by inter­molecular N—H⋯Cl hydrogen bonds.

## Related literature

For related structures, see: Bujak & Zaleski (1999 ▶); Feng *et al.* (2007 ▶); Chen (2009 ▶). For puckering parameters, see: Cremer & Pople (1975 ▶).
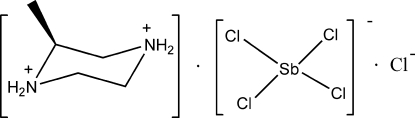


## Experimental

### 

#### Crystal data

(C_5_H_14_N_2_)[SbCl_4_]Cl
*M* *_r_* = 401.18
Orthorhombic, 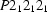

*a* = 7.745 (5) Å
*b* = 10.773 (7) Å
*c* = 16.318 (9) Å
*V* = 1361.6 (14) Å^3^
*Z* = 4
Mo *K*α radiation
μ = 2.97 mm^−1^
*T* = 293 K
0.28 × 0.26 × 0.22 mm

#### Data collection

Rigaku SCXmini diffractometer
Absorption correction: multi-scan (*CrystalClear*; Rigaku, 2005 ▶) *T*_min_ = 0.8, *T*_max_ = 0.9
13665 measured reflections
3086 independent reflections
3003 reflections with *I* > 2σ(*I*)
*R*_int_ = 0.026

#### Refinement

*R*[*F* ^2^ > 2σ(*F* ^2^)] = 0.019
*wR*(*F* ^2^) = 0.042
*S* = 1.08
3086 reflections
119 parameters
H-atom parameters constrained
Δρ_max_ = 0.90 e Å^−3^
Δρ_min_ = −0.33 e Å^−3^
Absolute structure: Flack (1983 ▶), 1299 Friedel pairs
Flack parameter: −0.037 (17)

### 

Data collection: *CrystalClear* (Rigaku, 2005 ▶); cell refinement: *CrystalClear*; data reduction: *CrystalClear*; program(s) used to solve structure: *SHELXS97* (Sheldrick, 2008 ▶); program(s) used to refine structure: *SHELXL97* (Sheldrick, 2008 ▶); molecular graphics: *SHELXTL* (Sheldrick, 2008 ▶); software used to prepare material for publication: *SHELXL97*.

## Supplementary Material

Crystal structure: contains datablocks I, global. DOI: 10.1107/S1600536810047689/bx2322sup1.cif

Structure factors: contains datablocks I. DOI: 10.1107/S1600536810047689/bx2322Isup2.hkl

Additional supplementary materials:  crystallographic information; 3D view; checkCIF report

## Figures and Tables

**Table 1:** Hydrogen-bond geometry (Å, °)

*D*—H⋯*A*	*D*—H	H⋯*A*	*D*⋯*A*	*D*—H⋯*A*
N1—H1*E*⋯Cl5^i^	0.90	2.29	3.190 (3)	179
N2—H2*B*⋯Cl5^ii^	0.90	2.27	3.150 (3)	166

Symmetry codes: (i) 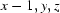; (ii) 
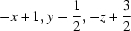
.

**Fig. 1.**
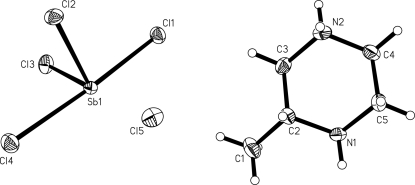
A view of (I) with atom labels. Displacement ellipsoids were drawn at the 30% probability level.

**Fig. 2.**
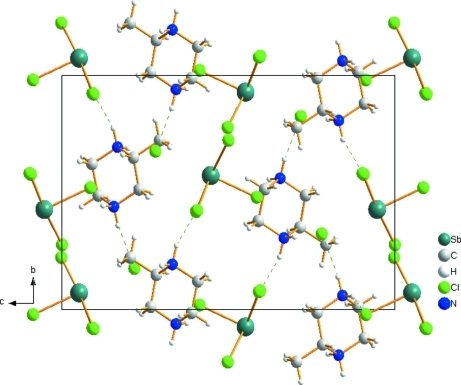
Packing diagram.

## supplementary crystallographic information

### Comment

Rencently, the crystal structure of some halogenoantimonate salts has been reported (Feng *et al.*, 2007; Bujak & Zaleski, 1999; Chen, 2009).The construction of new members of this family is important in the development of modern coordination chemistry. We report here the crystal structure of the title compound. In the complex anion of the title compound, *C*_5_H_14_N_2_. SbCl_4_.Cl, the Sb atom is tetracoordinate and has a saw-horse geometry.The cation complex adopt chair conformation with Cremer & Pople (1975) puckering parameters: Q_T_ = 0.556 (3) Å,

θ = 1.8 (3) ° , φ = 97 (14)°. The crystal structure is stabilized by two intermolecular N—H···Cl hydrogen bonds, Table 1.

### Experimental

A mixture of (*R*)-2-Methylpiperazine (2 mmol, 0.2 g), SbCl_3_(2 mmol, 0.46 g) and 10% aqueous HCl (20 ml) were mixed and dissolved in 10 ml water by heating to 353 K (0.5 h) forming a clear solution. The reaction mixture was cooled slowly to room temperature, crystals of the title compound were formed after 13 days.

### Refinement

All H atoms were placed in calculated positions, with C—H = 0.93–0.98Å and N—H = 0.90 Å, and re?ned using a riding model, with *U*_iso_(H)=1.2*U*_eq_(C,*N*) or 1.5 *U*_eq_(C) for methyl H atoms.

### Crystal data

**Table:**

(C_5_H_14_N_2_)[SbCl_4_]Cl	*F*(000) = 776
*M**_r_* = 401.18	*D*_x_ = 1.957 Mg m^−^^3^
Orthorhombic, *P*2_1_2_1_2_1_	Mo *K*α radiation, λ = 0.71073 Å
Hall symbol: P 2ac 2ab	Cell parameters from 3003 reflections
*a* = 7.745 (5) Å	θ = 2.9–27.5°
*b* = 10.773 (7) Å	µ = 2.97 mm^−^^1^
*c* = 16.318 (9) Å	*T* = 293 K
*V* = 1361.6 (14) Å^3^	Block, colorless
*Z* = 4	0.28 × 0.26 × 0.22 mm

### Data collection

**Table:**

Rigaku SCXmini diffractometer	3086 independent reflections
Radiation source: fine-focus sealed tube	3003 reflections with *I* > 2σ(*I*)
graphite	*R*_int_ = 0.026
Detector resolution: 13.6612 pixels mm^-1^	θ_max_ = 27.5°, θ_min_ = 2.9°
ω scans	*h* = −10→10
Absorption correction: multi-scan (*CrystalClear*; Rigaku, 2005)	*k* = −13→14
*T*_min_ = 0.8, *T*_max_ = 0.9	*l* = −21→21
13665 measured reflections	

### Refinement

**Table:**

Refinement on *F*^2^	Secondary atom site location: difference Fourier map
Least-squares matrix: full	Hydrogen site location: inferred from neighbouring sites
*R*[*F*^2^ > 2σ(*F*^2^)] = 0.019	H-atom parameters constrained
*wR*(*F*^2^) = 0.042	*w* = 1/[σ^2^(*F*_o_^2^) + (0.0207*P*)^2^] where *P* = (*F*_o_^2^ + 2*F*_c_^2^)/3
*S* = 1.08	(Δ/σ)_max_ = 0.003
3086 reflections	Δρ_max_ = 0.90 e Å^−^^3^
119 parameters	Δρ_min_ = −0.33 e Å^−^^3^
0 restraints	Absolute structure: Flack (1983), 1283 Friedel pairs
Primary atom site location: structure-invariant direct methods	Flack parameter: −0.037 (17)

### Special details

**Table:**

Geometry. All e.s.d.'s (except the e.s.d. in the dihedral angle between two l.s. planes) are estimated using the full covariance matrix. The cell e.s.d.'s are taken into account individually in the estimation of e.s.d.'s in distances, angles and torsion angles; correlations between e.s.d.'s in cell parameters are only used when they are defined by crystal symmetry. An approximate (isotropic) treatment of cell e.s.d.'s is used for estimating e.s.d.'s involving l.s. planes.
Refinement. Refinement of *F*^2^ against ALL reflections. The weighted *R*-factor *wR* and goodness of fit *S* are based on *F*^2^, conventional *R*-factors *R* are based on *F*, with *F* set to zero for negative *F*^2^. The threshold expression of *F*^2^ > σ(*F*^2^) is used only for calculating *R*-factors(gt) *etc*. and is not relevant to the choice of reflections for refinement. *R*-factors based on *F*^2^ are statistically about twice as large as those based on *F*, and *R*- factors based on ALL data will be even larger.

### Fractional atomic coordinates and isotropic or equivalent isotropic displacement parameters (Å^2^)

**Table:**

	*x*	*y*	*z*	*U*_iso_*/*U*_eq_	
Sb1	0.83387 (2)	0.572289 (14)	0.552350 (9)	0.02499 (5)	
C1	0.3416 (5)	0.7218 (3)	0.70580 (19)	0.0533 (8)	
H1A	0.2382	0.7109	0.6742	0.080*	
H1B	0.4391	0.6931	0.6748	0.080*	
H1C	0.3562	0.8082	0.7185	0.080*	
C2	0.3278 (4)	0.6485 (2)	0.78422 (15)	0.0306 (5)	
H2	0.4326	0.6617	0.8167	0.037*	
C3	0.3079 (4)	0.5122 (3)	0.76750 (17)	0.0380 (7)	
H3A	0.2117	0.4997	0.7304	0.046*	
H3B	0.4114	0.4817	0.7407	0.046*	
C4	0.1252 (4)	0.4866 (3)	0.8900 (2)	0.0479 (8)	
H4A	0.1130	0.4402	0.9406	0.058*	
H4B	0.0214	0.4743	0.8577	0.058*	
C5	0.1455 (5)	0.6213 (3)	0.90911 (17)	0.0428 (7)	
H5A	0.2425	0.6329	0.9459	0.051*	
H5B	0.0424	0.6515	0.9363	0.051*	
Cl1	0.59794 (9)	0.41872 (8)	0.59619 (5)	0.03962 (16)	
Cl2	1.05446 (8)	0.42321 (8)	0.59260 (5)	0.03824 (16)	
Cl3	0.82868 (11)	0.47961 (7)	0.41391 (4)	0.04005 (15)	
Cl4	1.08779 (11)	0.72263 (7)	0.49885 (5)	0.04645 (19)	
Cl5	0.84080 (12)	0.67609 (6)	0.71841 (4)	0.04331 (16)	
N1	0.1748 (3)	0.69351 (19)	0.83223 (13)	0.0326 (5)	
H1D	0.1906	0.7739	0.8451	0.039*	
H1E	0.0799	0.6883	0.8006	0.039*	
N2	0.2777 (3)	0.4395 (2)	0.84360 (15)	0.0375 (5)	
H2A	0.3721	0.4439	0.8757	0.045*	
H2B	0.2606	0.3593	0.8305	0.045*	

### Atomic displacement parameters (Å^2^)

**Table:**

	*U*^11^	*U*^22^	*U*^33^	*U*^12^	*U*^13^	*U*^23^
Sb1	0.02595 (8)	0.02336 (8)	0.02566 (7)	0.00076 (7)	0.00008 (7)	0.00201 (6)
C1	0.0526 (18)	0.063 (2)	0.0443 (16)	−0.003 (2)	0.0064 (18)	0.0194 (15)
C2	0.0271 (12)	0.0357 (14)	0.0289 (12)	−0.0029 (13)	−0.0017 (13)	0.0037 (10)
C3	0.0417 (18)	0.0405 (16)	0.0317 (14)	0.0067 (14)	0.0011 (14)	−0.0054 (11)
C4	0.046 (2)	0.0357 (17)	0.062 (2)	0.0015 (13)	0.0161 (16)	0.0157 (14)
C5	0.054 (2)	0.0423 (16)	0.0323 (14)	0.0061 (15)	0.0106 (15)	0.0039 (12)
Cl1	0.0315 (3)	0.0423 (4)	0.0450 (4)	−0.0033 (3)	0.0028 (3)	0.0054 (3)
Cl2	0.0314 (3)	0.0346 (4)	0.0488 (4)	0.0052 (3)	−0.0072 (3)	0.0052 (4)
Cl3	0.0360 (3)	0.0527 (4)	0.0314 (3)	−0.0035 (4)	0.0012 (4)	−0.0102 (3)
Cl4	0.0452 (4)	0.0438 (5)	0.0504 (4)	−0.0103 (4)	0.0059 (3)	−0.0038 (4)
Cl5	0.0481 (4)	0.0430 (4)	0.0389 (3)	0.0054 (4)	−0.0081 (4)	−0.0019 (3)
N1	0.0381 (12)	0.0246 (11)	0.0350 (11)	0.0031 (11)	−0.0027 (12)	0.0009 (8)
N2	0.0374 (12)	0.0264 (12)	0.0486 (13)	0.0024 (10)	−0.0033 (11)	−0.0007 (11)

### Geometric parameters (Å, °)

**Table:**

Sb1—Cl2	2.4351 (12)	C3—H3B	0.9700
Sb1—Cl3	2.4702 (14)	C4—N2	1.492 (4)
Sb1—Cl1	2.5667 (13)	C4—C5	1.493 (4)
Sb1—Cl4	2.6932 (13)	C4—H4A	0.9700
C1—C2	1.507 (4)	C4—H4B	0.9700
C1—H1A	0.9600	C5—N1	1.493 (3)
C1—H1B	0.9600	C5—H5A	0.9700
C1—H1C	0.9600	C5—H5B	0.9700
C2—N1	1.501 (4)	N1—H1D	0.9000
C2—C3	1.502 (4)	N1—H1E	0.9000
C2—H2	0.9800	N2—H2A	0.9000
C3—N2	1.486 (4)	N2—H2B	0.9000
C3—H3A	0.9700		
			
Cl2—Sb1—Cl3	89.51 (4)	N2—C4—C5	110.7 (3)
Cl2—Sb1—Cl1	89.95 (5)	N2—C4—H4A	109.5
Cl3—Sb1—Cl1	89.02 (4)	C5—C4—H4A	109.5
Cl2—Sb1—Cl4	88.38 (5)	N2—C4—H4B	109.5
Cl3—Sb1—Cl4	87.62 (4)	C5—C4—H4B	109.5
Cl1—Sb1—Cl4	176.26 (3)	H4A—C4—H4B	108.1
C2—C1—H1A	109.5	C4—C5—N1	110.3 (2)
C2—C1—H1B	109.5	C4—C5—H5A	109.6
H1A—C1—H1B	109.5	N1—C5—H5A	109.6
C2—C1—H1C	109.5	C4—C5—H5B	109.6
H1A—C1—H1C	109.5	N1—C5—H5B	109.6
H1B—C1—H1C	109.5	H5A—C5—H5B	108.1
N1—C2—C3	109.2 (3)	C5—N1—C2	113.0 (2)
N1—C2—C1	109.3 (3)	C5—N1—H1D	109.0
C3—C2—C1	111.4 (2)	C2—N1—H1D	109.0
N1—C2—H2	109.0	C5—N1—H1E	109.0
C3—C2—H2	109.0	C2—N1—H1E	109.0
C1—C2—H2	109.0	H1D—N1—H1E	107.8
N2—C3—C2	112.3 (2)	C3—N2—C4	111.7 (2)
N2—C3—H3A	109.1	C3—N2—H2A	109.3
C2—C3—H3A	109.1	C4—N2—H2A	109.3
N2—C3—H3B	109.1	C3—N2—H2B	109.3
C2—C3—H3B	109.1	C4—N2—H2B	109.3
H3A—C3—H3B	107.9	H2A—N2—H2B	107.9

### Hydrogen-bond geometry (Å, °)

**Table:**

*D*—H···*A*	*D*—H	H···*A*	*D*···*A*	*D*—H···*A*
N1—H1E···Cl5^i^	0.90	2.29	3.190 (3)	179
N2—H2B···Cl5^ii^	0.90	2.27	3.150 (3)	166

Symmetry codes: (i) *x*−1, *y*, *z*; (ii) −*x*+1, *y*−1/2, −*z*+3/2.
